# Combining genomic analyses with tumour-derived slice cultures for the characterization of an *EGFR*-activating kinase mutation in a case of glioblastoma

**DOI:** 10.1186/s12885-018-4873-9

**Published:** 2018-10-11

**Authors:** Lea Loriguet, Mony Chenda Morisse, Julie Dremaux, Louison Collet, Christophe Attencourt, Alexandre Coutte, Mathieu Boone, Henri Sevestre, Antoine Galmiche, Brigitte Gubler, Bruno Chauffert, Stephanie Trudel

**Affiliations:** 1 EA4666, LNPC, Université de Picardie Jules Verne, Amiens, France; 2 Laboratoire d’Oncobiologie moléculaire, Centre Hospitalier Universitaire Amiens-Picardie, Amiens, France; 3 Service d’Oncologie médicale, Centre Hospitalier Universitaire Amiens-Picardie, Amiens, France; 4 Service d’Anatomie et de cytologie pathologiques, Centre Hospitalier Universitaire Amiens-Picardie, Amiens, France; 5 Service d’Oncologie radiothérapique, Centre Hospitalier Universitaire Amiens-Picardie, Amiens, France; 6 Laboratoire de Biochimie, Centre Hospitalier Universitaire Amiens-Picardie, Amiens, France

**Keywords:** Glioblastoma, EGFR, Activating kinase mutation, Tyrosine kinase inhibitors, Afatinib, Next-generation sequencing, Tumour-derived slice cultures

## Abstract

**Background:**

Epidermal growth factor receptor (*EGFR*) gene alterations and amplification are frequently reported in cases of glioblastoma (GBM). However, *EGFR*-activating mutations that confer proven sensitivity to tyrosine kinase inhibitors (TKIs) in lung cancer have not yet been reported in GBM.

**Case presentation:**

Using next-generation sequencing, array comparative genomic hybridization and droplet digital PCR, we identified the p.L861Q *EGFR* mutation in a case of GBM for the first time. The mutation was associated with gene amplification. L861Q may be a clinically valuable mutation because it is known to sensitize non-small-cell lung cancers to treatment with the second-generation EGFR TKI afatinib in particular. Furthermore, we used slice culture of the patient’s GBM explant to evaluate the tumour’s sensitivity to various EGFR-targeting drugs. Our results suggested that the tumour was not intrinsically sensitive to these drugs.

**Conclusions:**

Our results highlight (i) the value of comprehensive genomic analyses for identifying patient-specific, targetable alterations, and (ii) the need to combine genomic analyses with functional assays, such as tumour-derived slice cultures.

**Electronic supplementary material:**

The online version of this article (10.1186/s12885-018-4873-9) contains supplementary material, which is available to authorized users.

## Background

Glioblastoma (GBM) is the most common malignant, primary brain tumour. This type of tumour is extremely invasive and difficult to treat, and is characterized by intense and aberrant vascularization and high resistance to radiotherapy and chemotherapy. Standard treatment of GBM includes surgery and concomitant radiotherapy and temozolomide (TMZ), followed by systemic treatment with TMZ in an adjuvant setting [1]. At present, there are few treatment options in the event of relapse, and the clinical effectiveness is low [2]. The median survival time is still no longer than 15 months, with a 5-year survival rate of 4 to 5% [3].

The molecular identification and characterization of the alterations that drive oncogenesis and the progression of GBM are important factors in the development of effective therapies. Glioblastoma was the first type of cancer to be studied by the Cancer Genome Atlas Research Network [4]. Thus, complex genetic alterations and genomic profiles (which usually involve multiple signalling pathways) have been defined for GBM; they include copy number variations (chr 7 gain, chr 10 and chr 9p losses), gene mutations and alterations (in *PTEN*, *TP53*, *EGFR*, and *PIK3CA*), gene amplifications or deletions (*EGFR*, *MDM2*, *PDGFRA*, and *CDKN2A/B*) and promoter methylation (in *MGMT*) [4, 5]. *EGFR* is frequently affected by many different RNA and DNA alterations; aberrant *EGFRvIII* transcripts [6] and *EGFR* gene amplification are the most frequently observed genomic abnormalities. *EGFR* point mutations are infrequent and they tend to cluster in the extracellular domain [4, 5]. Thus, the so-called activating mutations reported in lung cancers (which more commonly harbour kinase domain alterations) have not been reported in GBM. Here, we describe (i) the identification and characterization of an *EGFR*-activating kinase mutation in a case of GBM, and (ii) the clinical response to afatinib after relapse.

## Methods

### DNA extraction from tumour and plasma samples

Genomic DNA was obtained from formalin-fixed, paraffin-embedded (FFPE) tissue by automated extraction with the Tissue Preparation System and the VERSANT® Tissue Preparation Reagents kit (Siemens). For plasma DNA, 10 ml of blood were collected in cell-free DNA BCT CE tubes (Streck). Plasma was separated from the cellular fraction by centrifugation (10 min, 2000 *g*, at room temperature, twice) and stored at − 80 °C. Before extraction, plasma samples were centrifuged (10 min, 13,000 *g*, at 4 °C), and plasma DNA was extracted with the QIAmp Circulating Nucleic Acid Kit (Qiagen). The concentration of DNA from FFPE tissue or plasma was determined using the Qubit dsDNA HS Assay Kit (ThermoFisher).

### DNA copy number analysis

Array comparative genomic hybridization (aCGH) experiments were performed according to the manufacturer’s instructions (Agilent Technologies) after optimization for DNA obtained from FFPE tissue samples (FFPE DNA). Briefly, 500 ng of FFPE DNA labelled with Cy3-dCTP or Cy5-dCTP was competitively hybridized with heat-fragmented female reference genomic DNA on 8 × 60 K CGH microarrays (Sureprint G3 Human). The arrays were washed and scanned, and the images were obtained using Feature Extraction software (Agilent Technologies). The scanned data were analyzed with Cytogenomics v.4.0 software (Agilent Technologies). The ADM-2 algorithm and a threshold value of 6.0 were applied, along with appropriate filters. Gains and deletions of chromosomal regions were considered when (i) the corresponding plotted oligoprobes presented an absolute log ratio ≥ 0.25, and (ii) the minimum size of region for a gain/deletion was ≥1000 kb. Gene amplification was considered when the plotted oligoprobes targeting this gene had a log ratio ≥ 0.25.

### Panel-based next-generation sequencing

Next-generation sequencing (NGS) was performed with Ion AmpliSeq technology (Life Technologies), according to the manufacturer’s instructions. Sequencing libraries were prepared using the Colon and Lung Cancer Research v2 community panel (Life Technologies) and a custom-designed complementary panel (WG_IAD123003.20170703). The target genes and exons are listed in Additional file 1: Table S1. Multiplex barcoded libraries were generated with Ion AmpliSeq Library kit v2 (using 10 ng of FFPE DNA as the input) and normalized to 50 pmol, using the Ion Library TaqMan quantification kit. The pooled barcoded libraries were processed on an Ion Chef™ System (using an Ion 520 & Ion 530 Kit-Chef) and sequenced on an Ion S5™ Instrument (using an Ion 530 Chip Kit). The FASTQ format sequencing data were processed and aligned with the human genome (hg19) using the Ion Torrent Suite v5.0. Ion Reporter v5.0 (Life Technologies) was used for variant calling. The sequences of interest were then visualized with the Integrative Genomics Viewer (Broad Institute).

### Droplet-based digital PCR

The *EGFR* c.2582 T > A (p.L861Q) mutation was detected by allelic discrimination with TaqMan probes and primers (Life Technologies). The droplet-based digital PCR (ddPCR) assay of plasma DNA and FFPE DNA has been described and validated elsewhere [7]. Using genomic DNA extracted from wild type (WT) and mutated cell lines, the limit of blank and the limit of detection were found to be 0 and 3 droplets, respectively. The probe bearing the fluorescein (FAM) fluorophore (λ_ex_ = 494 nm / λ_em_ = 518 nm) was specific for the WT allele, while the probe bearing the VIC fluorophore (λ_ex_ = 538 nm / λ_em_ = 554 nm) hybridized specifically to the mutant allele. One μl of 25X Drop Stabilizer (RainDance Technologies) and 0.625 μl of 40X TaqMan SNP assay reagent (C_172767645_10) were mixed with 12.5 μL of 2X TaqMan Genotyping Master Mix (Life Technologies), in a final reaction volume of 25 μl. For FFPE DNA, 10 to 40 ng were used per reaction. For plasma DNA, a volume of 10.88 μl was used - regardless of the amount of DNA. Next, 5 pl droplets were prepared with the RainDrop® Source (RainDrop® Digital PCR System, RainDance Technologies). Emulsions were collected in eight-strip PCR tubes (Axigen). The samples were thermally cycled (using the standard conditions recommended by RainDance) with a C1000 touch thermal cycler (BioRad®). Lastly, samples were sealed with opaque flat caps (RainDance Technologies) and transferred into the RainDrop® Sense instrument. After all the samples had been evaluated, data from cluster plots were spectrally compensated and analyzed using RainDrop Analyst software, according to standard procedures. Two samples from mutation-bearing patients were used as controls, in order to define gates around the cluster displaying a signal for WT copies (FAM fluorescence) and mutant copies (VIC fluorescence). These gates were applied to all samples. Each series included a positive control and a “no template” (negative) control.

### Short-term, ex vivo culture

Pieces of surgically resected GBM underwent short-term culture, as previously described [8]. Briefly, about 1 cm^3^ of non-necrotic tumour was selected by the pathologist, and tumour samples were prepared as 300 μm thick slices with a vibrating blade microtome (VT1200S Vibratome, Leica). The slices were placed in 24-well plates and cultured for 48 h in Dulbecco’s Modified Eagle Medium culture medium (supplemented with 10% fetal calf serum (PAN-Biotech), penicillin/streptomycin, and 1% glutamine) at 37 C in a 5% CO_2_ atmosphere. Cetuximab (Merck-Serono), erlotinib, and afatinib (both from Euromedex) were selected on the basis of their activity against the EGFR. The drug concentrations in the culture medium were as follows: 30 μM for cetuximab, 1 μM for erlotinib, and 10 μM for afatinib. These single drug concentrations were chosen according to our previous study [8]. Cetuximab was dissolved in saline, and erlotinib and afatinib were dissolved in DMSO. All three stock solutions were stored at − 20 °C prior to use.

### Cell proliferation index

Tumour slices were fixed in formalin and then paraffin-embedded; 3 μm sections were cut and stained with hematoxylin-phloxin-saffron (HPS) reagent, in order to select non-necrotic and non-fibrotic areas with a high density of tumour cells (as defined by a senior pathologist (C.A.)). Two tumour slices were analyzed for each experimental condition, and ten microscope images were recorded for each slide. Hence, a total of 20 images (representing at least 1000 tumour cells in all) were analyzed for each experimental condition. The monoclonal antibody MIB1 (Immunotech) was used to immunostain Ki67. The cell proliferation index (CPI) was determined as the ratio of Ki67-positive tumour cells (evaluated visually by a senior pathologist) to total tumour cells (i.e. after the exclusion of cells from the matrix and vessels). The mean CPI was calculated using the results from all slides.

### Western blots

After processing as described above, tumour slices were frozen at − 20 °C for subsequent immunoblotting. Total extracts were prepared as described previously, loaded onto polyacrylamide gels for SDS-PAGE, and then transferred to nitrocellulose membranes [8]. Antibodies against extracellular regulated kinase 1/2 (ERK) and phosphorylated ERK1/2 (P-ERK) were purchased from Cell Signaling Technology. Antibodies against β-actin were purchased from Sigma. Secondary antibodies coupled to horse radish peroxidase were obtained from GE Healthcare. An enhanced chemiluminescence reaction was used for detection. The immunoblots were scanned and quantified using Image J software (National Institutes of Health).

### Statistical analysis

All statistical analyses were performed with Prism 5 software (GraphPad Software). The threshold for statistical significance (in two-tailed *t*-test) was set to *p* < 0.05.

## Case presentation

In March 2016, a 71-year-old female of Caucasian origin was referred to Amiens-Picardie University Medical Center (Amiens, France) for gait impairment. Magnetic resonance imaging (MRI) showed a voluminous mass in the right temporal lobe, the features of which were strongly suggestive of GBM. The patient underwent subtotal resection. A histopathologic study of FFPE surgical samples submitted in toto revealed a dense proliferation of highly atypical tumor cells. Many atypical mitotic figures were observed. Angiogenesis had produced large glomeruloid vascular channels. These morphological features were highly subjective of GBM. The tumor cells were labelled by antibodies against GFAP and Olig2 but not by an antibody against isocitrate dehydrogenase 1 (IDH1)-R132H. A diagnosis of IDH-WT glioblastoma was made, and the *MGMT* promoter was found to be unmethylated. Following our observation of an *EGFR* mutation, a complementary immunohistochemical study was performed in order to rule out a diagnosis of bronchopulmonary carcinoma: the tumor cells did not expresss polyclonal AE1/AE3 cytokeratin, CK7, NapsinA, TTF1 or P40. After the patient has provided her written, informed consent, she was enrolled in the Bi-GlAM study (designed to evaluate plasma DNA in GBM patients during their clinical follow-up).

### Identification of an EGFR-activating mutation associated with gene amplification

The panel-based NGS mutational profile revealed several SNPs and one somatic mutation (Table 1). The tumour did not present any *IDH 1* (exon 4) or *IDH 2* (exon 4) mutations, prompting a histomolecular diagnosis of *IDH*-WT glioblastoma. The c.2582 T > A substitution in the *EGFR* gene was of particular interest; it resulted in an amino acid change at position 861 from leucine (Leu, L) to glutamine (Gln, Q). Hence, a p.L861Q mutation (COSM6213) was unambiguously identified. The allele frequency was 17% (*T* = 0.8308 and A = 0.1692; Table 1). However, this ratio was not consistent with the tissue’s tumor cell content, as evaluated by the pathologist (around 80%). When comparing the results for samples from the same run, we found that the number of reads for each *EGFR* exon was much higher in the patient’s tumour (Additional file 2: Table S2; by around 10-fold for exons 12 and 21, and 15-fold for exons 18, 19 and 20). This finding strongly suggested the occurrence of *EGFR* gene amplification, which was later confirmed by the aCGH experiments.

**Table 1:** Characteristics of the variants identified in the tumour sample

Genes	Transcript	Coding	Amino Acid Change	PolyPhen	SIFT	Allele Coverage	Allele Ratio	dbSNP	Clinical significance
DDR2	NM_006182.2					C = 1051, *T* = 938	C = 0.5284, *T* = 0.4716	rs3738807	Benign
FGFR3	NM_001163213.1	c.1959G > A	p.(=)			G = 0, A = 1128	G = 0.0, A = 1.0	rs7688609	Likely benign
EGFR	NM_005228.3					A = 0, *T* = 3916	A = 0.0, *T* = 1.0	rs1558544	na
EGFR	NM_005228.3	c.2361G > A	p.(=)			G = 2, A = 3929	G = 5.0E-4, A = 0.9995	rs1050171	Likely benign
EGFR	NM_005228.3	c.2582 T > A	p.Leu861Gln	0.999	0.01	*T* = 3304, A = 673	T = 0.8308, A = 0.1692	rs121913444	Pathogenic

**Fig. 1:**
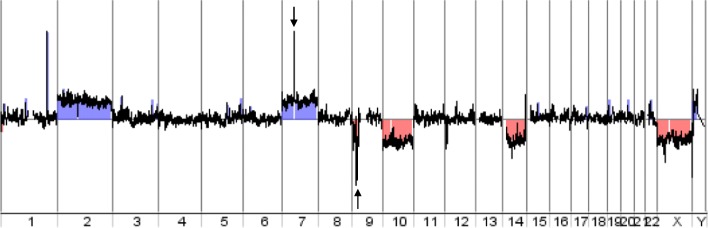
DNA copy number analysis. The genome view generated from aCGH data showed detectable aberrations in the DNA extracted from FFPE primary tumour: gain of chromosomes 2 and 7, loss of the short arm of chromosome 9, and loss of chromosomes 10, 14 and X. The aCGH data also revealed amplification of the q32.1 region (chr 1) and the *EGFR* gene (chr 7, black arrow), together with a homozygous deletion of the *CDKN2A* gene (chr 9, black arrow). *aCGH: array comparative genomic hybridization; chr: chromosome*

As shown in Fig. 1, the tumor presented a loss of chromosome 10 (mean log ratio: − 0.446) and a partial loss of the short arm of chromosome 9 (9p24.1-p21.2, mean log ratio: − 0.385) including the homozygous deletion of CDKN2A gene (arrow, mean log ratio: − 1.155) and a gain in chromosome 7 (mean log ratio: 0.376) including the amplification of *EGFR* gene (arrow, mean log ratio: 3.642). The combination of tri/polysomy of chromosome 7 and loss of heterozygosity of 10q are characteristic molecular features in GBM - especially when they are associated with *EGFR* amplification [9]. Additional chromosomal aberrations could be observed, such as gain of chromosome 2 (mean log ratio: 0,367), gain of chromosome X (mean log ratio: 0.398), amplification of the *MDM4* gene (1q32.1, mean log ratio: 3.681) and loss of the short arm of chromosome 14 (mean log ratio: − 0.416).

The NGS and aCGH data all confirmed the *EGFR* gene amplification, and suggested that the p.L861Q mutation was not present in all *EGFR* alleles (since the observed allele ratio was only 17% for the mutation).

**Fig. 2:**
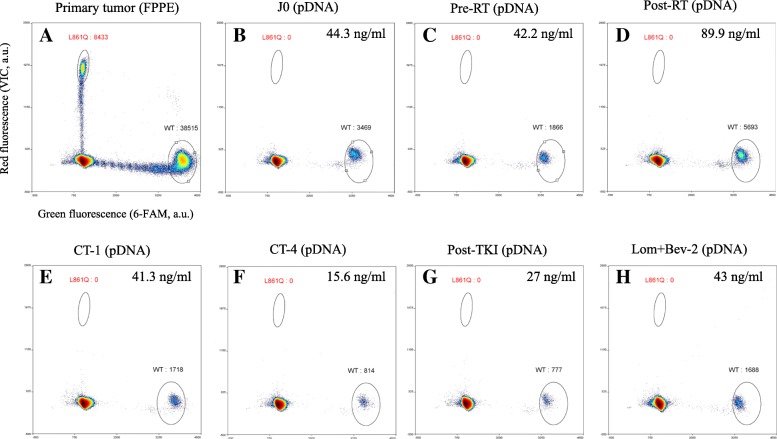
L861Q TaqMan assay (droplet digital PCR). 2D plots and amounts of L861Q-positive droplets (indicated in red) obtained via ddPCR with FFPE DNA (**a**) or cfDNA (**b** to **h**). **a**) the primary tumour, **b**) on the day of surgery (D0), **c**) before concomitant chemotherapy (CT) and radiotherapy (RT) (pre-RT), **d**) 1 month after RT (post-RT), **e**) 1 month after the first cycle of CT (CT-1), **f**) 1 month after four cycles of CT (CT-4), **g**) after 9 weeks of afatinib treatment (post-TKI), and **h**) after two cycles of lomustine with bevacizumab (Lom + Bev-2). The plasma DNA concentration is expressed as the amount per ml of plasma. *ddPCR, droplet digital PCR; FFPE, formalin-fixed, paraffin-embedded; pDNA, plasma DNA; AU, arbitrary units; WT, wild type; CT, chemotherapy; RT, radiotherapy*

The presence of this unusual mutation in a case of GBM was confirmed by ddPCR using a TaqMan assay to discriminate between the WT and p.L861Q alleles. As shown in Fig. 2a, the mutation was detected in the FFPE DNA sample from the primary tumour with an allele frequency of 18% (8433 out of 46,948 droplets).

### Treatment decisions and radiological analysis

**Fig. 3:**
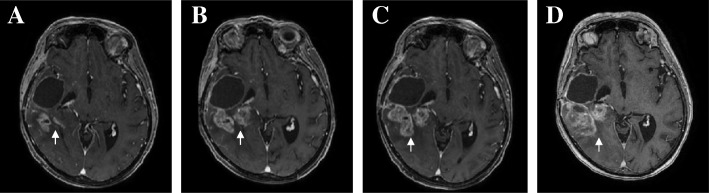
Magnetic resonance imaging. Gadolinium-contrast-enhanced T1-weighted 3D magnetic resonance imaging of the brain after radiotherapy (**a**), before afatinib treatment (**b**), and after 4 weeks (**c**) and 8 weeks (**d**) of afatinib treatment. The lesion is indicated by an arrow

The first-line treatment comprised subtotal resection of the lesion (in March 2016), a combination of radiotherapy and TMZ, and then adjuvant TMZ [1]. After four cycles of TMZ, however, disease progression (according to the Revised Assessment in Neuro-Oncology criteria, RANO [10]) was observed on MRI (Fig. 3b). Given the presence of the p.L861Q *EGFR* mutation (known to confer sensitivity to second generation TKIs [11–13]), the patient started a second-line course of off-label treatment with afatinib after the provision of fully informed consent. A radiological disease assessment after 1 month of afatinib treatment (40 mg/day) did not reveal any significant lesion growth (i.e. stable disease; Fig. 3c). The occurrence of asthenia prompted us to reduce the dose of afatinib to 30 mg/day. A month later, MRI revealed an increase in contrast enhancement (i.e. disease progression; Fig. 3d). We withdrew the TKI at this point, and initiated third-line treatment with bevacizumab and lomustine. The patient died in July, 2017.

### Longitudinal monitoring of the L861Q mutation in plasma DNA

In an attempt to monitor the tumour response and to detect recurrent disease during clinical surveillance, a specific ddPCR assay was used to assay circulating DNA from plasma samples for the L861Q-mutation. The plasma samples were collected before concomitant radiotherapy/TMZ, 1 month after radiotherapy, 1 month after the first cycle of TMZ, 1 month after the fourth cycle of TMZ, after 9 weeks of afatinib treatment, and after two cycles of lomustine with bevacizumab, and assayed for the presence of the p.L861Q mutation. The plasma DNA concentration for each sample is indicated in Fig. 2. However, the p.L861Q mutation was not detected at any of the monitoring time points.

### Inhibition of cell proliferation and EGFR pathway in tumour explants

**Fig. 4:**
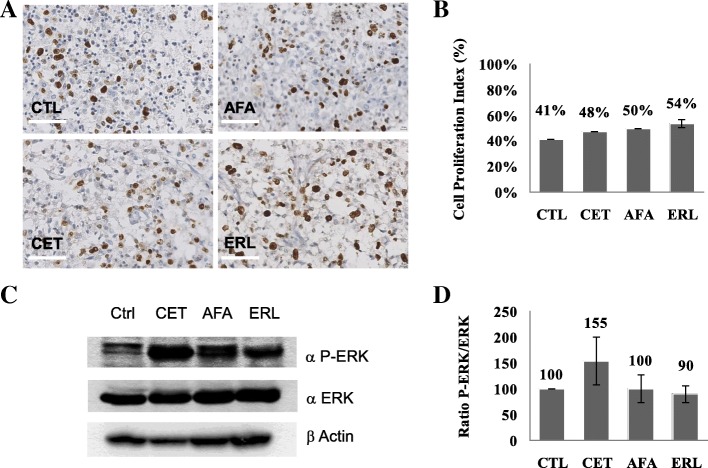
Inhibition of cell proliferation and the EGFR pathway in tumour explants. **a**) Microscope images (× 40) of Ki67 immunostaining on tumour slices from the patient’s glioblastoma for each condition: control (CTL), cetuximab (CET), afatinib (AFA), and erlotinib (ERL). **b**) Visual quantification of the proportion of Ki67-positive cells. There were no statistically significant differences between CTL on one hand and CET (*p* = 0.45), AFA (*p* = 0.6) and ERL (*p* = 0.37). **c**) Immunoblot of tumour slices treated for 48 h under four conditions: CTL, CET, AFA and ERL. To investigate the drugs’ efficacy, antibodies against phospho-ERK (P-ERK) and ERK (ERK) were applied, and actin was used as a loading control. **d**) Quantification of the P-ERK/ERK ratio, as a percentage of the control P-ERK/ERK ratio. There were no statistically significant differences between the negative control on one hand and cetuximab (*p* = 0.36), afatinib (*p* = 0.99) and erlotinib (*p* = 0.59) on the other. Values are expressed as the mean (*n* = 4) and the standard error of the mean (represented by error bars)*.* The scale bar corresponds to 25 μm

Given that (i) EGFR alterations are the most frequent genomic defects in GBM, and (ii) a number of targeted therapies are on the market, we performed short-term cultures of neurosurgical tumour resections with the EGFR-targeting drugs cetuximab, erlotinib and afatinib. Each drug’s effect was evaluated as the percentage of Ki67-positive tumour cells in an immunochemical assay and a visual assessment. There were no statistically significant differences between the negative control on one hand and cetuximab (*p* = 0.45), afatinib (*p* = 0.6) and erlotinib (*p* = 0.37) on the other (Fig. 4a and b) - suggesting that all three molecules would have been poorly active against the patient’s tumour.

We also used immunoblotting to assess expression levels of the markers ERK and phospho-ERK (pERK) in samples obtained by short-term culture. The pERK/ERK ratio (reflecting the activation of the RAF-MEK-ERK pathway) was calculated by densitometric analysis of the immunoblot (Fig. 4c and d). When compared with a negative control, neither cetuximab, afatinib nor erlotinib was associated with a statistically significant difference in the pERK/ERK ratio (*p* = 0.36, 0.99 and 0.59, respectively).

## Discussion and conclusions

We used panel-based NGS and ddPCR to identify the exon 21 p.L861Q *EGFR* mutation for the first time in a case of *IDH*-WT GBM. This somatic point mutation was associated with *EGFR* gene amplification, as revealed by NGS and aCGH experiments (Table 1 and Fig. 1). As reported by other researchers, most *EGFR* point mutations are accompanied by regional DNA amplification [4, 5]. It is highly probable that the two oncogenic alterations (the p.L861Q mutation and the *EGFR* amplification, both of which are known to be driver alterations) were present in all tumor cells; the cells all had the mutated allele and the *EGFR* amplification, although the amplified allele was not mutated. Although the *EGFR* gene is often amplified in GBM, kinase mutations in these tumours are rare – which explains the lack of significant benefit of EGFR TKI treatment in GBM relative to lung cancers, which are more likely to harbour kinase-domain EGFR alterations.

In non-small cell lung cancer (NSCLC), *EGFR* mutations in exons 18 to 21 can be used to predict the effectiveness of EGFR TKIs [14, 15]. However, the exon 21 p.L861Q mutation is rarer (frequency: 0.9–3.2%) than the classical exon 19 deletions (frequency: 46–59%) and the exon 21 p.L858R point mutation (25–38%) [13–16]. L861Q may be a sensitizing mutation [17, 18], since it confers particular sensitivity to the second-generation EGFR TKI afatinib (relative to first-generation TKIs like gefitinib and erlotinib) [11–13].

There are very few published clinical data on the effectiveness of afatinib in patients with GBM. A recent Phase I/II study of afatinib (as a monotherapy or combined with TMZ) in recurrent GBM found that certain selected patient populations (including those with high levels of *EGFRvIII* immunoreactivity, *EGFR* amplification, or *PTEN* loss) had a promising treatment response and extended progression-free survival [19]. However, the mutational status of *EGFR* was not investigated in this cohort. In view of (i) the very limited choice of second-line treatments with sustained clinical benefit in the context of relapsed GBM, (ii) the presence of the p.L861Q sensitizing mutation and the concomitant *EGFR* amplification, and (iii) the promising results of the Phase I/II study in selected patient populations, we decided to initiate treatment with afatinib. However, no clinical benefits could be observed after 9 weeks of treatment; imaging and clinical assessments indicated first stable disease and then disease progression (Fig. 3).

One can argue that the blood-brain barrier (BBB) makes the central nervous system a sanctuary site [20, 21]. It has been shown that despite their low molecular weight, both erlotinib and gefitinib seem to reach limited concentrations in the cerebrospinal fluid (CSF) when compared with the blood plasma [22, 23]. In contrast, data on efficacy in patients with brain metastases who were treated with afatinib after chemotherapy and an EGFR-TKI were recently published [24]. Over 70% of the patients with brain metastases had either partial response or stable disease after treatment with afatinib; brain responses were documented in 35% of the patients, and 76% of patients did not develop new metastases. The observed brain metastasis responses provide clinical evidence that the CSF concentration of afatinib is enough to inhibit tumour growth. A subgroup analysis of the LUX-Lung 3 trial has also confirmed the efficacy of first-line afatinib treatment in central nervous system metastases [25].

In order to explore the tumour’s inherent biological sensitivity to afatinib in our patient, we tested the samples generated from short-term cultures of surgical specimens. This technique is a simple, robust method for evaluating the effects of targeted therapies on fresh human tumours, i.e. in conditions that resemble the clinical setting as closely as possible [8]. Tumour fragments were exposed to relevant concentrations of cetuximab, erlotinib and afatinib - all of which target the EGFR-RAF-MEK-ERK transduction pathway. As shown in Fig. 4, proliferation was not inhibited by any of the drugs, and inhibition of ERK phosphorylation was never observed. These results suggest that the tumour was not intrinsically sensitive to afatinib, and that the BBB and bioavailability have a minor role in the lack of clinical response observed in our patient. In the present case, the absence of a treatment response is probably due (at least in part) to the low frequency of the L861Q mutant allele conferring sensitivity to afatinib. Furthermore, several clinical studies have shown that the response to oncogene-driven targeted therapies does not solely depend on the presence of actionable alterations [26, 27]. The tumour’s genomic landscape and histology are also major determinants of sensitivity to targeted therapies, as oncogenic transduction pathways may be regulated differently.

In addition to the obvious potential therapeutic value of the p.L861Q mutation, we also hypothesize that the mutation could be used as a plasma molecular biomarker for disease monitoring. The analysis of tumour-derived cfDNA in a patient’s plasma has an increasingly valuable role in early diagnosis, prognosis, the treatment response, and real-time disease monitoring in different types of cancer [28–30]. Since tumour DNA levels in blood are low, highly sensitive ddPCR is increasingly being used to detect rare mutational targets. However, our ddPCR assays did not detect the p.L861Q mutation at any time point during the clinical follow-up - even after relapse (Fig. 2). This absence was probably related to the very low amount of cfDNA obtained from plasma samples. The low amount of blood cfDNA in GBM might explain why very few genomic alterations can be detected in plasma derived DNA from brain tumours (relative to gastrointestinal or lung cancers) [31, 32]. In fact, recent publications have demonstrated that CSF is more suitable than plasma for the characterization of genomic alteration in brain tumours in general and GBMs in particular [33, 34]. Given the intended frequency of sample collection, it was not ethical or feasible to collect cfDNA from CSF via lumbar puncture on a routine basis.

In conclusion, the implementation of NGS in clinical molecular biology laboratories and the development of comprehensive panels for the combined exploration of multiple cancers (NSCLC, colorectal cancer, melanoma, gastro-intestinal stromal tumours and glioma) can lead to the fortuitous identification of molecular aberrations that may confer sensitivity to targeted therapies. Since the genetic profile alone does not always predict a tumour’s drug sensitivity, it may be particularly relevant to combine the molecular data with short-term tumour slice cultures exposed to selected targeted therapies. This approach might be particularly appropriate in GBM because the standard of care for newly diagnosed patients usually includes neurosurgery and thus the availability of tumour tissue.

## Additional files

Additional file 1: **Table S1.** The list of target genes and exons. (PPT 107 kb)

Additional file 2: **Table S2.** The number of reads per amplicon obtained with panel-based, next-generation sequencing. The number of reads for EGFR exons corresponding to the patient’s tumour is given in red type. (PPT 120 kb)

## Abbreviations

BBB: Blood-brain barrier
cfDNA: Cell-free DNA
CGH: Comparative genomic hybridization
CPI: Cell proliferation index
CSF: Cerebrospinal fluid
ddPCR: Droplet-based digital PCR
EGFR: Epidermal growth factor receptor
ERK: Extracellular regulated kinase
FFPE: Formalin-fixed, paraffin-embedded
GBM: Glioblastoma
HPS: Hematoxylin-phloxin-saffron
IDH: Isocitrate dehydrogenase
MRI: Magnetic resonance imaging
NGS: Next-generation sequencing
NSCLC: Non-small cell lung cancer
TKI: Tyrosine kinase inhibitor
TMZ: Temozolomide
WT: Wild type
