# Supplementary material for: Combining genomic analyses with tumour-derived slice cultures for the characterization of an EGFR-activating kinase mutation in a case of glioblastoma
Source: BMC Cancer. 2018 Oct 11;18:964. doi: 10.1186/s12885-018-4873-9 (PMC6180520; doi:10.1186/s12885-018-4873-9)
Supplement: Supplementary file 1 — Table S1. The list of target genes and exons. (PPT 107 kb) [file 12885_2018_4873_MOESM1_ESM.ppt]

## Slide 1
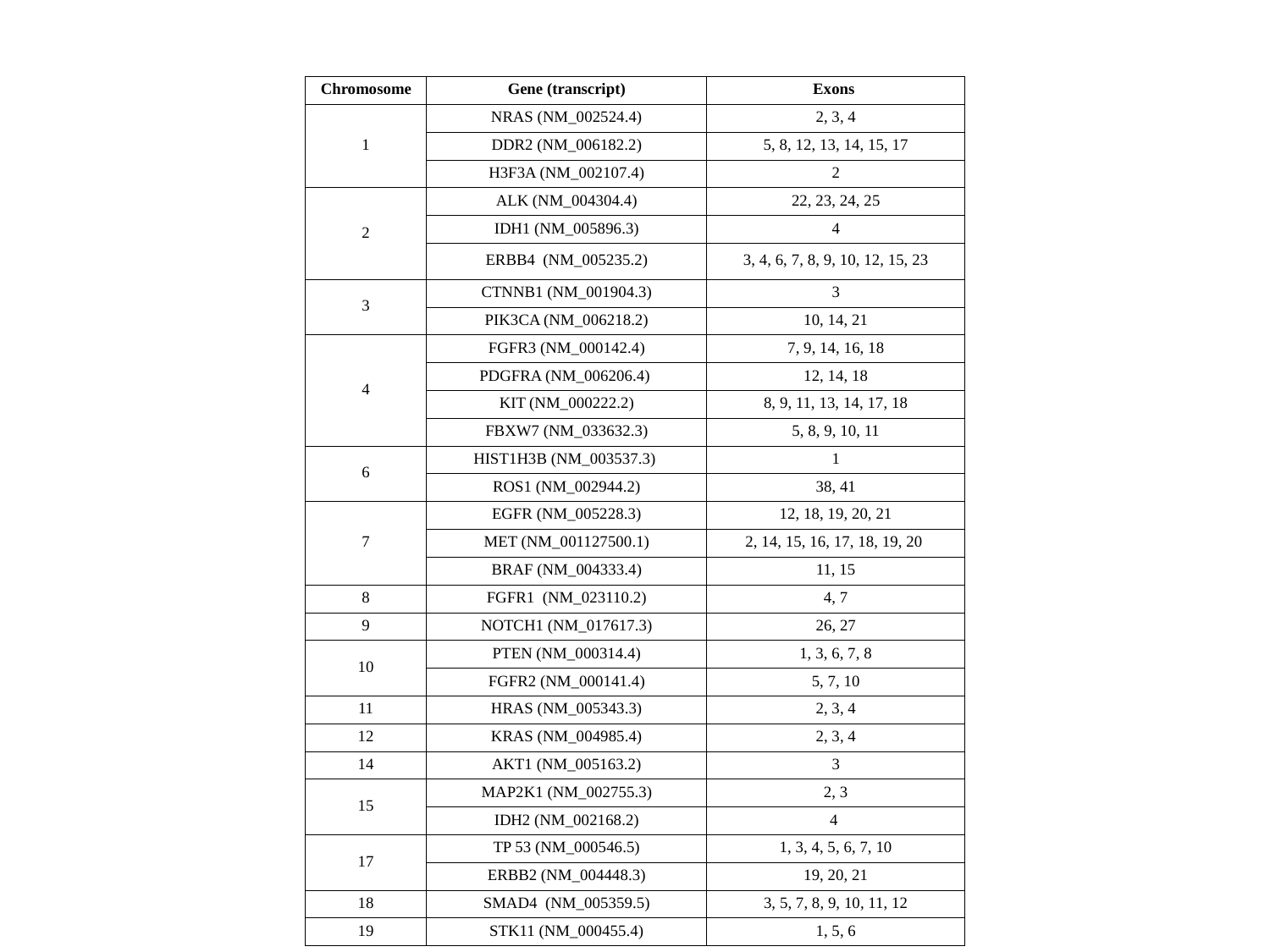

| Chromosome | Gene (transcript) | Exons |
| --- | --- | --- |
| 1 | NRAS (NM\_002524.4) | 2, 3, 4 |
| | DDR2 (NM\_006182.2) | 5, 8, 12, 13, 14, 15, 17 |
| | H3F3A (NM\_002107.4) | 2 |
| 2 | ALK (NM\_004304.4) | 22, 23, 24, 25 |
| | IDH1 (NM\_005896.3) | 4 |
| | ERBB4 (NM\_005235.2) | 3, 4, 6, 7, 8, 9, 10, 12, 15, 23 |
| 3 | CTNNB1 (NM\_001904.3) | 3 |
| | PIK3CA (NM\_006218.2) | 10, 14, 21 |
| 4 | FGFR3 (NM\_000142.4) | 7, 9, 14, 16, 18 |
| | PDGFRA (NM\_006206.4) | 12, 14, 18 |
| | KIT (NM\_000222.2) | 8, 9, 11, 13, 14, 17, 18 |
| | FBXW7 (NM\_033632.3) | 5, 8, 9, 10, 11 |
| 6 | HIST1H3B (NM\_003537.3) | 1 |
| | ROS1 (NM\_002944.2) | 38, 41 |
| 7 | EGFR (NM\_005228.3) | 12, 18, 19, 20, 21 |
| | MET (NM\_001127500.1) | 2, 14, 15, 16, 17, 18, 19, 20 |
| | BRAF (NM\_004333.4) | 11, 15 |
| 8 | FGFR1 (NM\_023110.2) | 4, 7 |
| 9 | NOTCH1 (NM\_017617.3) | 26, 27 |
| 10 | PTEN (NM\_000314.4) | 1, 3, 6, 7, 8 |
| | FGFR2 (NM\_000141.4) | 5, 7, 10 |
| 11 | HRAS (NM\_005343.3) | 2, 3, 4 |
| 12 | KRAS (NM\_004985.4) | 2, 3, 4 |
| 14 | AKT1 (NM\_005163.2) | 3 |
| 15 | MAP2K1 (NM\_002755.3) | 2, 3 |
| | IDH2 (NM\_002168.2) | 4 |
| 17 | TP 53 (NM\_000546.5) | 1, 3, 4, 5, 6, 7, 10 |
| | ERBB2 (NM\_004448.3) | 19, 20, 21 |
| 18 | SMAD4 (NM\_005359.5) | 3, 5, 7, 8, 9, 10, 11, 12 |
| 19 | STK11 (NM\_000455.4) | 1, 5, 6 |
