# Supplementary material for: Combining genomic analyses with tumour-derived slice cultures for the characterization of an EGFR-activating kinase mutation in a case of glioblastoma
Source: BMC Cancer. 2018 Oct 11;18:964. doi: 10.1186/s12885-018-4873-9 (PMC6180520; doi:10.1186/s12885-018-4873-9)
Supplement: Supplementary file 2 — Table S2. The number of reads per amplicon obtained with panel-based, next-generation sequencing. The number of reads for EGFR exons corresponding to the patient’s tumour is given in red type. (PPT 120 kb) [file 12885_2018_4873_MOESM2_ESM.ppt]

## Slide 1
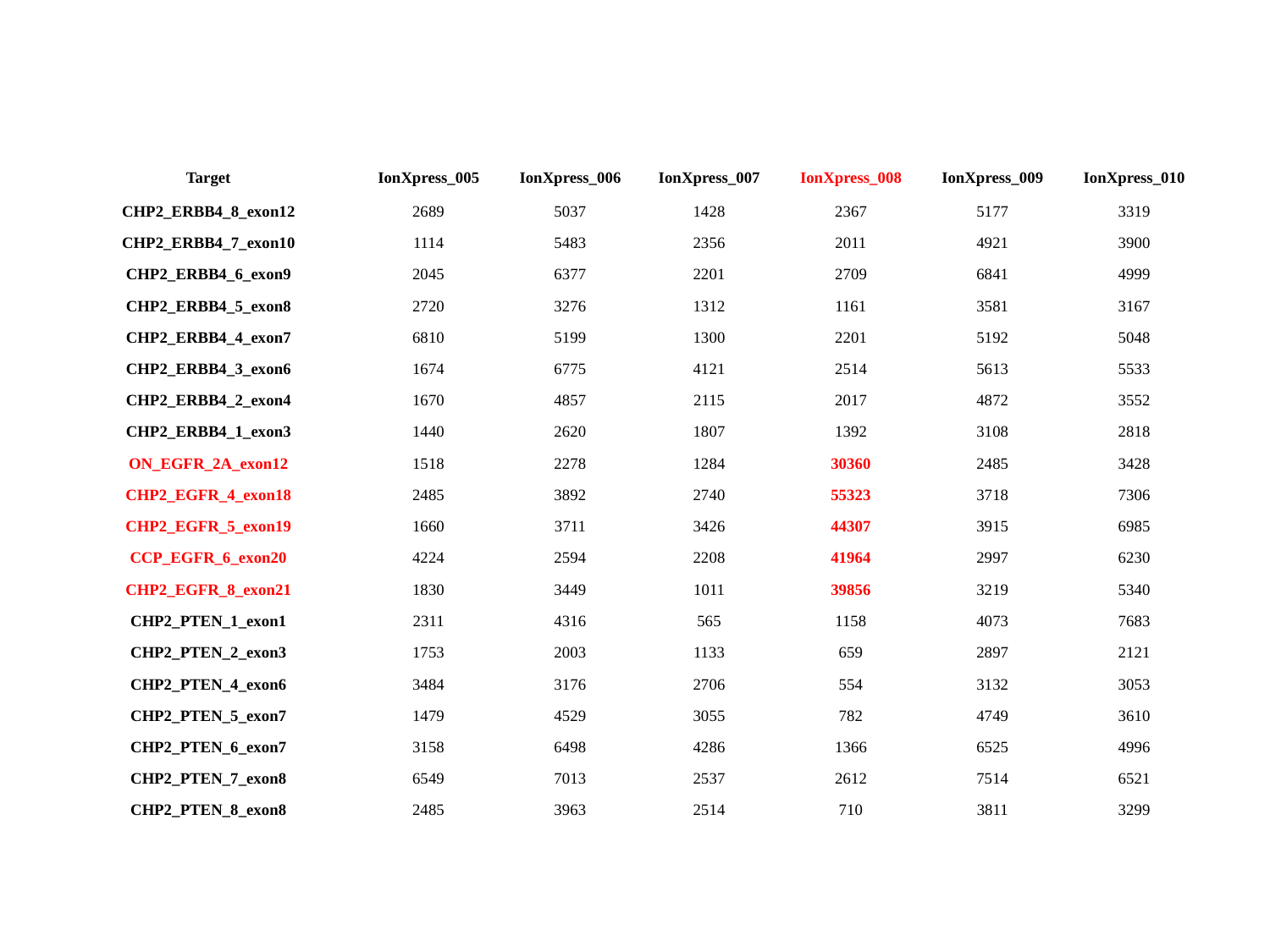

| Target | IonXpress\_005 | IonXpress\_006 | IonXpress\_007 | IonXpress\_008 | IonXpress\_009 | IonXpress\_010 |
| --- | --- | --- | --- | --- | --- | --- |
| CHP2\_ERBB4\_8\_exon12 | 2689 | 5037 | 1428 | 2367 | 5177 | 3319 |
| CHP2\_ERBB4\_7\_exon10 | 1114 | 5483 | 2356 | 2011 | 4921 | 3900 |
| CHP2\_ERBB4\_6\_exon9 | 2045 | 6377 | 2201 | 2709 | 6841 | 4999 |
| CHP2\_ERBB4\_5\_exon8 | 2720 | 3276 | 1312 | 1161 | 3581 | 3167 |
| CHP2\_ERBB4\_4\_exon7 | 6810 | 5199 | 1300 | 2201 | 5192 | 5048 |
| CHP2\_ERBB4\_3\_exon6 | 1674 | 6775 | 4121 | 2514 | 5613 | 5533 |
| CHP2\_ERBB4\_2\_exon4 | 1670 | 4857 | 2115 | 2017 | 4872 | 3552 |
| CHP2\_ERBB4\_1\_exon3 | 1440 | 2620 | 1807 | 1392 | 3108 | 2818 |
| ON\_EGFR\_2A\_exon12 | 1518 | 2278 | 1284 | 30360 | 2485 | 3428 |
| CHP2\_EGFR\_4\_exon18 | 2485 | 3892 | 2740 | 55323 | 3718 | 7306 |
| CHP2\_EGFR\_5\_exon19 | 1660 | 3711 | 3426 | 44307 | 3915 | 6985 |
| CCP\_EGFR\_6\_exon20 | 4224 | 2594 | 2208 | 41964 | 2997 | 6230 |
| CHP2\_EGFR\_8\_exon21 | 1830 | 3449 | 1011 | 39856 | 3219 | 5340 |
| CHP2\_PTEN\_1\_exon1 | 2311 | 4316 | 565 | 1158 | 4073 | 7683 |
| CHP2\_PTEN\_2\_exon3 | 1753 | 2003 | 1133 | 659 | 2897 | 2121 |
| CHP2\_PTEN\_4\_exon6 | 3484 | 3176 | 2706 | 554 | 3132 | 3053 |
| CHP2\_PTEN\_5\_exon7 | 1479 | 4529 | 3055 | 782 | 4749 | 3610 |
| CHP2\_PTEN\_6\_exon7 | 3158 | 6498 | 4286 | 1366 | 6525 | 4996 |
| CHP2\_PTEN\_7\_exon8 | 6549 | 7013 | 2537 | 2612 | 7514 | 6521 |
| CHP2\_PTEN\_8\_exon8 | 2485 | 3963 | 2514 | 710 | 3811 | 3299 |
